# Physiotherapist or physician as primary assessor for patients with suspected knee osteoarthritis in primary care – a cost-effectiveness analysis of a pragmatic trial

**DOI:** 10.1186/s12891-022-05201-3

**Published:** 2022-03-17

**Authors:** Chan-Mei Ho-Henriksson, Mikael Svensson, Carina A Thorstensson, Lena Nordeman

**Affiliations:** 1 Region Västra Götaland, Primary Care Rehabilitation, Närhälsan Lidköping Rehabmottagning, Lidköping, Sweden; 2 Department of Health and rehabilitation, Unit of Physiotherapy, University of Gothenburg, Sahlgrenska Academy, Institute of Neuroscience and Physiology, Gothenburg, Sweden; 3 University of Gothenburg, Sahlgrenska Academy, Institute of Medicine, School of Public Health and Community Medicine, Gothenburg, Sweden; 4 Research and Development Department at Region Halland, Halmstad, Sweden; 5 Region Västra Götaland, Research, Education, Development and Innovation, Primary Health Care, Research, Education, Development and Innovation Centre Södra Älvsborg, Borås, Sweden

**Keywords:** Physiotherapist, Primary care, Cost-efficiency, Knee osteoarthritis, Health care process, Direct access, Triage

## Abstract

**Background:**

Over the next decade, the number of osteoarthritis consultations in health care is expected to increase. Physiotherapists may be considered equally qualified as primary assessors as physicians for patients with knee osteoarthritis. However, economic evaluations of this model of care have not yet been described. To determine whether physiotherapists as primary assessors for patients with suspected knee osteoarthritis in primary care are a cost-effective alternative compared with traditional physician-led care, we conducted a cost-effectiveness analysis alongside a randomized controlled pragmatic trial.

**Methods:**

Patients were randomized to be assessed and treated by either a physiotherapist or physician first in primary care. A cost-effectiveness analysis compared costs and effects in quality adjusted life years (QALY) for the different care models. Analyses were applied with intention to treat, using complete case dataset, and missing data approaches included last observation carried forward and multiple imputation. Non-parametric bootstrapping was conducted to assess sampling uncertainty, presented with a cost-effectiveness plane and cost-effectiveness acceptability curve.

**Results:**

69 patients were randomized to a physiotherapist (*n* = 35) or physician first (*n* = 34). There were significantly higher costs for physician visits and radiography in the physician group (*p* < 0.001 and *p* = 0.01). Both groups improved their health-related quality of life 1 year after assessment compared with baseline. There were no statistically significant differences in QALYs or total costs between groups. The incremental cost-effectiveness ratio for physiotherapist versus physician was savings of 24,266 €/lost QALY (societal perspective) and 15,533 €/lost QALY (health care perspective). There is a 72–80% probability that physiotherapist first for patients with suspected knee osteoarthritis is less costly and differs less than ±0.1 in QALY compared to traditional physician-led care.

**Conclusion:**

These findings suggest that physiotherapist-led care model might reduce health care costs and lead to marginally less QALYs, but confidence intervals were wide and overlapped no difference at all. Health consequences depending on the profession of the first assessor for knee osteoarthritis seem to be comparable for physiotherapists and physicians. Direct access to physiotherapist in primary care seems to lead to fewer physician consultations and radiography. However, larger clinical trials and qualitative studies to evaluate patients’ perception of this model of care are needed.

**Clinical trial registration:**

The study was retrospectively registered in clinicaltrial.gov, ID: NCT03822533.

**Supplementary Information:**

The online version contains supplementary material available at 10.1186/s12891-022-05201-3.

## Background

Osteoarthritis (OA) is the leading cause of chronic musculoskeletal pain worldwide and ranked as one of the highest contributors to global disability [1, 2]. Patients with knee OA (KOA) rate among the lowest in health-related quality of life (HrQoL) compared with patients suffering from other chronic musculoskeletal disorders [3]. Patients with OA suffer from more comorbidities [4] and have a higher risk of all-cause mortality than the general population [5]. Arthritis is one of the most common reasons for primary care visits [6] and the OA consultation rate in Swedish health care is expected to increase the coming decade [7]. Recommended core treatments for patients with KOA are patient education, exercise, and if necessary, weight reduction [8–11]. Non-surgical treatments delay or prevent the need of surgery up to 7 years among more than 50% of patients with KOA [12]. Most patients with OA can successfully be managed in primary care without surgery. Although the number of physicians is growing, there is still a shortage of physicians in Swedish primary care [13]. A similar trend of escalation of OA consultations and lack of physicians in primary care has been seen even in other countries [14, 15].

A more sustainable model of care, where patients get evidence-based non-surgical OA treatment faster, could be physiotherapists as primary assessors. Direct access to physiotherapists has been implemented in many countries already [16]. Several systematic reviews have reported about direct access to physiotherapist, which is suggested to be safe, more effective and less costly care model for patients with musculoskeletal disorders in primary care, community care, hospital, outpatient clinics and occupational clinics [17–19]. The results are similar when focusing specifically on direct access to physiotherapists in primary care [20]. Many patients believe that a physician consultation is required before physiotherapist assessment and treatment [21]. Also, former experiences of physician assessments and expectations of further investigations such as radiography are other reasons for consulting physician first [22]. Despite different assessment strategies, where physiotherapists perform a musculoskeletal examination and physicians in addition have access to imaging when diagnosing KOA, there is high diagnostic agreement between physiotherapists and orthopaedic surgeons or sports medicine physicians for knee pain disorders including KOA [23]. As radiography is unnecessary in diagnosing patients with typical presentation of OA [24], both physicians and physiotherapists can assess patients with suspected KOA. Physiotherapists and orthopaedic surgeons have similar high diagnostic accuracy compared to magnetic resonance imaging of musculoskeletal disorders (75% respectively 81%), and significantly higher accuracy than family practice and internal medicine physicians who have an accuracy of 35% [25]. Moreover, Samsson et al. [26] concluded that physiotherapists and orthopaedic surgeons determined similar diagnoses, and recommended similar management for patients with musculoskeletal disorders.

Mean total costs are 10–20% lower for direct access to physiotherapists compared to physician-led care for patients with musculoskeletal disorders [19, 27–30]. Non-surgical treatments account for one fifth of all OA-related costs, including 4% for physiotherapy [31]. For patients who also suffer from comorbidities, the costs increase with the number of comorbidities [32]. Systematic reviews report that physiotherapist as primary assessor appears to be a cost-effective management for patients with musculoskeletal disorders, yet there is a need for more randomized controlled studies [17–20, 33]. Due to the heterogeneity of musculoskeletal disorders included in the reviews, and lack of presenting specific diseases, it is unclear if these costs also applies to patients with KOA which is a long-lasting disease with long treatment periods.

This study is based on a clinical trial evaluating the impact on HrQoL in patients with KOA when physiotherapists and physicians act as primary assessors. The results implied that both providers are equally qualified as primary assessors [34]. In this study, we are interested in how the first treatment period of early-stage KOA is affected from an economic perspective by different primary assessors. For this purpose, we evaluated the cost-effectiveness from a societal and health care perspective 1 year after first assessment, comparing the intervention physiotherapist as primary assessor with traditional physician-led care in primary care for patients with KOA.

## Methods

### Study design

A cost-effectiveness analysis (CEA) alongside an assessor-blinded randomized controlled pragmatic trial, which has been described elsewhere [34]. Primary assessment, diagnosis, and treatment either by a physiotherapist or a physician in primary care were compared. The study was conducted in primary care in southwestern Sweden during 2013–2017. The societal perspective and a health care perspective were applied in the CEA. The societal perspective investigates the welfare effects, and includes all resources, which in this study are individual resources (productivity loss) and health care resources. The societal perspective was considered as the primary perspective since it includes the full set of identified cost consequences. The health care perspective represents the payer’s perspective and consists of cost items in the health care sector. The study was retrospectively registered (29/01/2019) in clinicaltrial.gov, ID: NCT03822533. The Swedish Ethical Review Authority approved the study, reference number: 979–12. All participants provided written informed consent.

### Sample size

The power analysis was calculated for the clinical trial. Eighty percent power was achieved with 43 patients per group using a two-sided t-test with a minimal clinical difference of Euroqol 5 dimensions 3 levels questionnaire (EQ-5D-3L) index at 0.121 [35, 36], standard deviation (SD) 0.2 and a significance level set at *p* < 0.05. The dropout rate was estimated to be 14%, giving a total desired sample size of 100 patients, with 50 patients per group.

### Interventions

**Fig. 1:**
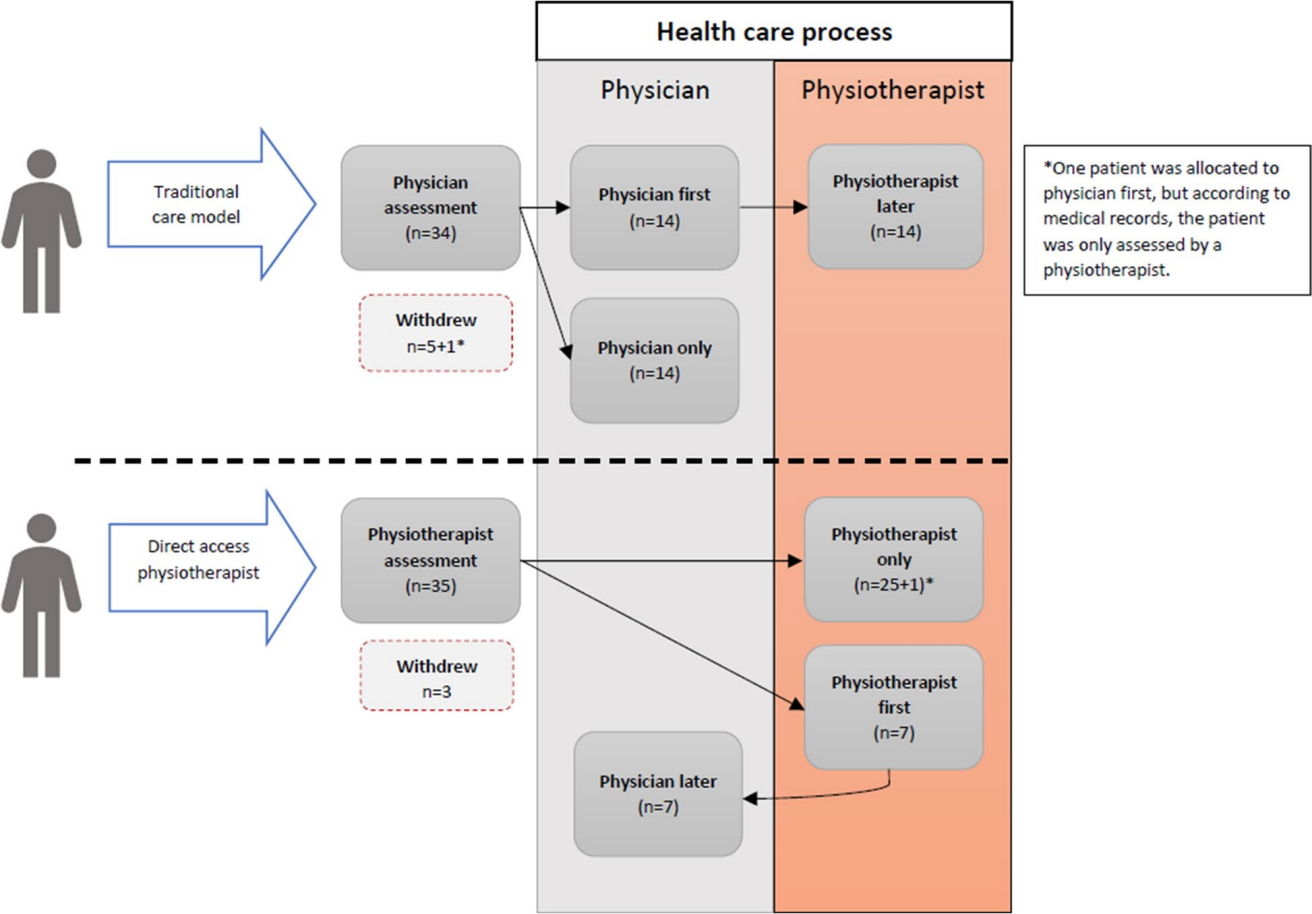
Health care pathways

Patients were randomized to be assessed and diagnosed either by physiotherapist or physician first. Due to the pragmatic setting, assessments, diagnostic tests and treatment plans varied. Usual physiotherapist assessments could comprise patient history, diagnostic tests (measuring joint range of motion, muscle strength in lower extremities), differential diagnostics to rule out other knee injuries, and functional assessment with physical performance tests. Physician assessment could be similar to the physiotherapist assessment, but also include radiography in the diagnostic evaluation. If needed, patients consulted the other health care provider any time after the first assessment (Fig. 1). Both groups’ treatment strategies were individually adapted based on national treatment guidelines [37]. Physiotherapy treatment involved individual and/or group treatment. The individual treatment consisted of information about KOA, introduction to an exercise program (physiotherapist-led or home exercising), pain treatment or walking aids. Group treatment was based on the Swedish national treatment program Better management of OA (BOA) [38]. The BOA-program comprised three sessions of patient education and 6 weeks of exercising either at the rehabilitation centre or at home. Physician treatment could include information, medical prescriptions, and referrals to radiography, a physiotherapist or another health care provider.

### Outcomes

Demographic data was assessed at baseline including age, gender, education level, current employment, pain duration and BMI (calculated with measured length and weight). Pain intensity was measured using a visual analogue scale (VAS) 0–100 mm [39] and physical function with the 30-s chair stand test [40].

#### Health outcomes

HrQoL was used as the generic measure for health improvement and was measured at baseline, 3-, 6- and 12-month follow-up. The time horizon of 1 year was chosen to evaluate the impact of different primary assessors and the first treatment period of early KOA. In general, treatment periods end within 6 months and thereafter the patients continue their rehabilitation through self-management. HrQoL was measured with EQ-5D-3L index - Swedish version [41, 42]. The index was calculated using the United Kingdom tariff [43] and ranges between − 0.594 to 1 where 1 indicates full health. For each participant, EQ-5D-3L index was used when calculating quality adjusted life years (QALY) [43] using linear interpolation between each measurement point and the trapezoidal rule to calculate the “*area under the curve*”.

#### Costs

The following data was extracted from medical records to calculate total health care costs: physiotherapist - visits and telephone calls, physician - visits including telephone calls, drug prescriptions, writing letters, nurse - visits and telephone calls, referrals to radiography, referrals to orthopaedic surgeons and sick leave periods. Physician and physiotherapist medical records were collected retrospectively after the last 12-month follow-up. Health care use and prescribed drugs were collected from the regional health care databases of Region Västra Götaland VEGA and Digitalis. VEGA included information on the individual level about inpatient and outpatient health care use, locations of the health care/rehabilitations centres, diagnoses and medical measures. Costs are presented in Euro (€), and annual average exchange rates were used for the Swedish Krona (SEK) for the study period (2013–2017). Data was collected from The Riksbank, Sweden’s central bank. Standard costs for primary care visits were used for the years 2013–2017 when the study was conducted. Clinical estimations from physiotherapists, physicians and nurses were used to calculate durations for telephone calls and other administrative patient related activities (drug prescriptions which were not included in the visit and writing letters about test results).

Inclusion criteria for data extraction from the drug database were prescribed drugs belonging to the Anatomical Therapeutic Chemical Classification groups M01 anti-inflammatory and anti-rheumatic products, M02 topical products for joint and muscular pain, M03 muscle relaxants, M09 other drugs for disorders of the musculoskeletal system, N02A opioids, N02B other analgesics and antipyretics. The following variables were obtained from the database: collected drugs, substance, strength, amount, total cost, and benefit cost and patients’ charge for the drugs.

Productivity loss were calculated with mean gross salary including social fees (31.42%) using data from the National Board of Health and Welfare for the area where the study was conducted. Productivity loss was valued with the human capital approach, which includes all work hours lost due to health problems and health care visits [44, 45]. Number of sick leave days were extracted from medical certificates in medical records. Productivity loss due to health care visits were based on presumed visiting times from clinical estimations: 45 min for a physiotherapist visit, 10 min for a physiotherapist call, 30 min for a physician visit, 15 min for a physician call, 15 min for a nurse visit, 20 min for a nurse call, 1 h for inpatient visits (radiography, orthopaedic surgeon), and plus 1 h for travel and waiting time for each visit. Net mean salary was used for unpaid work if the patient was on sick leave or retired.

### Statistical analysis

**Fig. 2:**
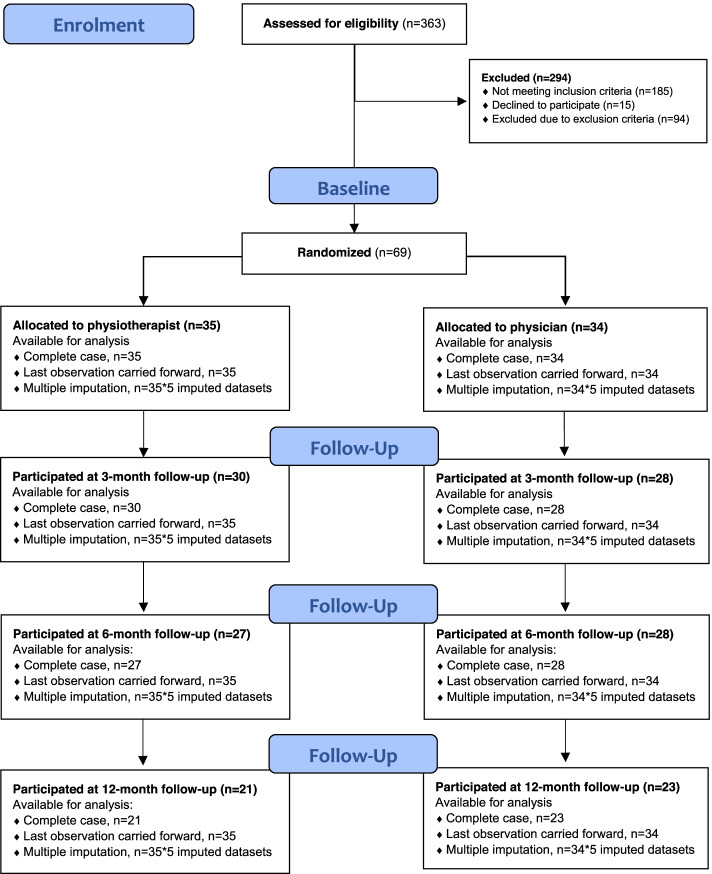
Flow chart of available cases for QALY analyses

Data was analysed descriptively and presented as numbers and percent, mean and SD or median and 25th to 75th percentiles. The analyses were applied with intention-to-treat where all the patients received the randomized allocated intervention – first assessment by either physiotherapist or physician. Analyses with this dataset including the follow-ups will be mentioned as complete case (CC). Comparative analyses with independent samples t-test and standard linear regression were used to analyse differences in mean costs and mean QALYs. These analyses are presented with 95% confidence interval (CI) and the significance level was set at *p* < 0.05. Regression analyses of mean QALYs were adjusted with baseline differences in EQ-5D-3L index [46]. Mean QALY calculations were analysed with three different datasets: CC, and datasets with imputed data for missing values of EQ-5D-3L index using imputations with last observation carried forward (LOCF) and multiple imputation (MI) (Fig. 2). We used two different approaches (LOCF and MI) to deal with missing data as a robustness check of the results. QALYs were recalculated in the new imputed datasets before performing comparative regression analyses.

Imputations with LOCF used the last observation of EQ-5D-3L index in each subject and imputed the value for every follow-up that had a missing value of this variable. MI was applied in Statistical Package for Social Science (SPSS) Windows, Version 25.0 [47], using linear regressions to generate random numbers for missing values of EQ-5D-3L index. Missing values were checked for random patterns. Imputed with following predictor variables: group, age, gender, BMI, education level, baseline value of pain intensity and 30-s chair stand test, and EQ-5D-3L index at baseline and all the follow-ups. The MI procedure created five different imputed datasets, which were used in the comparative regression analyses and resulted in output for each imputed dataset plus pooled results with estimation of what the results would have been if the original dataset had no missing values. These statistical analyses were performed in SPSS.

#### Cost-effectiveness analysis

The CEA compared costs and effects for the two alternatives (physiotherapist or physician as primary assessor) and were based on collected data from the clinical trial. The results are an incremental cost-effectiveness ratio (ICER), i.e. the ratio between the difference in mean costs and mean QALYs between the groups from baseline to the 12-month follow-up (∆Cost/∆QALYs). Costs and QALYs remain undiscounted due to the follow up being confined to 1 year [48].

#### Sampling uncertainty

Non-parametric bootstrapping was conducted to demonstrate the uncertainties surrounding the ICER. The results of the bootstrapping are presented in a cost-effectiveness plane (CE-plane) to illustrate the range of 200 bootstrap resampled ICERs and a 95% CI if applicable. The ICERs fall into one of four quadrants in the CE-plane; upper right quadrant/north east corner – the intervention costs more and has better effect than the control treatment; lower right quadrant/south east corner – costs less and better effect; lower left quadrant/south west corner – the intervention costs less and has less effect; upper left quadrant/north west corner – costs more and less effect. The cost-effectiveness acceptability curve (CEAC) illustrates the probability that the intervention group *“Physiotherapist as primary assessor”* is cost-effective compared with the control group *“Physician as primary assessor”*. The bootstrap analyses were performed in STATA 17 [49] with MI using “nearest neighbour matching” and with same predictor variables as mentioned above.

## Results

To determine if the care model with physiotherapist as primary assessor was more cost-effective than a physician as primary assessor for patients with suspected KOA, 35 patients were assessed by physiotherapist first and 34 patients by a physician first. Sixty-four percent completed the 12-month follow-up (40% (14/35) dropout in the physiotherapist group and 32% (11/34) in the physician group). Medical records, i.e. records of assessment or treatments for knee disorders, were available in 67% (46/69) for physiotherapist journals and 59% (41/69) for physician journals. Available data from regional databases were 59% (41/69), eight patients withdrew from the study and 20 patients did not reply when contacted for approval for further data extraction from the regional databases. Patient characteristics (Table 1) have been described earlier [34].

**Table 1:** Demographic features of the groups at baseline assessment

	Physiotherapist assessment (***n*** = 35)	Physician assessment (***n*** = 34)
	Mean (SD); median [25th to 75th percentile] or % (n)	Mean (SD); median [25th to 75th percentile] or % (n)
**Age (years)**	62 (12); 63 [52–71]	59 (12); 57 [48–68]
**Gender (females)**	60% (21/35)	68% (23/34)
**Level of education**		
Primary school (≤ 9 years)	23% (8/35)	12% (4/34)
Secondary school (10–12 years)	43% (15/35)	59% (20/34)
Tertiary school (> 12 years)	34% (12/35)	29% (10/34)
**Current employment**		
Employed/working	54% (19/35)	50% (17/34)
Working rate (%)	88 (4.7); 100 [81–100]	93 (4.2); 100 [100–100]
Unemployed	0% (0/35)	3% (1/34)
Retired/early retirement	43% (15/35)	38% (13/34)
Sick leave	3% (1/35)	6% (2/34)
**Pain duration (months)**	14 (22); 9 [3–12]	10 (16); 4 [2–11]
**BMI**^**a**^**(kg/m**^**2**^**)**	30 (4.4); 29 [26–31]	29 (6.7); 27 [25–31]
BMI: normal weight (18,5-24,9)	9% (3/35)	29% (10/34)
BMI: overweight (25–29,9)	54% (19/35)	38% (13/34)
BMI: obese (> 30)	37% (13/35)	32% (11/34)
**HrQoL (EQ-5D-3L)**^**b**^		
Index	0.73 (0.12); 0.73 [0.69–0.80]	0.62 (0.22); 0.73 [0.62–0.73]
**Pain intensity (VAS 0–100 mm)**^**c**^	45 (16); 47 [35–55]	52 (16); 51 [40–69]
**Physical function (30CST)**^**d**^	12 (4.6); 12 [9–14]	11 (3.3); 11 [8–13]

^a^Body Mass Index

^b^Health-related Quality of Life using Euroqol 5 dimension 3 Levels (EQ-5D-3L). Higher values indicate better health-related quality of life

^c^Visual analogue scale. Higher values indicate higher pain intensity

^d^30 seconds Chair Stand Test. Higher values indicate better physical function

### Health care visits

Most patients in this study consulted a physiotherapist (Fig. 1 and Table 2). The majority of the control group were referred to physiotherapy after a physician consultation. There were 26 patients who only had physiotherapy and 14 who only had physician consultations. Seven patients in the physiotherapist group consulted a physician as well, and 14 patients in the physician group sought a physiotherapist for further treatment. On average, patients in the physiotherapist group consulted a physiotherapist four times individually plus two group visits and had 0.3 physician visits. Patients who had a physician as primary assessor had on average 1.5 physician visits and four individual physiotherapist visits plus 1.5 group visits (Table 3). Patients in the physician group were more frequently referred to radiography than in the physiotherapist group (39% physician group, 9% physiotherapist group). Less than 10% were referred to an orthopaedic surgeon for further examination (physiotherapist group 6%, physician group 14%) (Table 2). Most patients who needed a physician consultation were managed by a nurse first via a telephone call (Table 3).

**Table 2:** Health care pathways and treatments

		Patient education (n)	Exercise therapy (n)	Referral physiotherapist (n)	Referral radiography (n)	Referral orthopaedic surgeon (n)	Prescription drugs (n)	Corticoid injections (n)	Sick leave (n)
**Physiotherapist assessment (*n*** **= 33)**^**a**^	**Physiotherapist only (*n*** **= 26)**^**a**^	12	25	0	0	0	1	0	0
	**Physiotherapist first, then physician (*n*** **= 7)**	4	7	3	3	2	3	1	0
**Physician assessment (*n*** **= 28)**^**b**^	**Physician only (*n*** **= 14)**	0	0	12	4	0	5	1	0
	**Physician first, then physiotherapist (*n*** **= 14)**	9	11	11	7	4	7	2	1
	**Total (*n*** **= 61)**	**25/61**	**43/61**	**26/61**	**14/61**	**6/61**	**16/61**	**4/61**	**1/61**

^a^Number of patients analysed. Three dropouts due withdrawal from the study after baseline assessment. One patient was added from the physician group

^b^One patient was allocated to physician first, but according to medical records, the patient was only assessed by a physiotherapist

**Table 3:** Total health care services in the groups

	Physiotherapist assessment (***n***^**a**^ = 32)		Physician assessment (***n*** = 29)	
	Sum	Mean (SD^**b**^)	Sum	Mean (SD)
**Physiotherapist**				
*Individual visits*	128	4.0 (4.7)	115	4.0 (9.4)
*Group visits*	67	2.1 (3.8)	41	1.5 (4.5)
*Telephone calls*	10	0.3 (0.6)	1	0.4 (0.2)
**Physician**				
*Individual visits*	8	0.3 (0.6)	41	1.5 (0.6)
*Telephone calls*	2	0.06 (0.4)	7	0.3 (0.6)
*Drug prescriptions only*	7	0.2 (0.8)	7	0.3 (0.6)
*Letter*	1	0.03 (0.2)	7	0.3 (0.5)
**Nurse**				
*Individual visits*	1	0.03 (0.2)	4	0.1 (0.6)
*Telephone calls*	17	0.6 (1.3)	32	1.1 (2.1)

^a^Number of participants

^b^Standard deviation

### Diagnosis, treatments and sick leave

Seventy percent received either an OA diagnosis according to the ICD 10-SE-diagnoses (M17 Gonarthrosis, M19 Arthrosis), or the assessor diagnosed with “gonarthrosis” or “arthrosis” in free text in the medical journal. The most common physiotherapy treatments were exercising (43/61). Fewer than half of all patients (25/61) received patient education about OA. About a third (10/34) received advice about non-prescription drugs and one in four patients received drug prescription (16/61) when consulting a physician (Table 2). More patients in the physician-first group received drug prescriptions than the physiotherapist group (physiotherapist group *n* = 4, physician group *n* = 12). Non-steroid anti-inflammatory drugs (Naproxen, Diclofenac or Ibuprofen) were most frequently prescribed. A few (4/61) patients were treated with corticoid injections. Three to 6 % self-reported a sick leave period, and one patient (1/61) required medical certificate for a longer sick leave period, with a total of 26 days of work absence (Table 2).

### Costs

The costs for the two groups using the societal perspective were 633 €/patient (SD 620) and 996 €/patient (SD 1276) for physiotherapist and physician group respectively. Total average costs from a health care perspective for the care model with physiotherapist as primary assessor were 515 €/patient (SD 541) and 748 €/patient (SD 885) with physician as primary assessor. The total cost differences were not statistically significant. However, there were significantly higher costs in the physician group compared with the physiotherapist group regarding physician visits, writing letters and radiography, and significantly higher telephone costs in the physiotherapist group compared with the physician group (Table 4). The mean difference in costs using the societal perspective was − 364 €/patient (CI: − 870 to143) in favour of the physiotherapist-first group and − 233 €/patient (CI: − 605 to 139) using the health care perspective (Table 5).

**Table 4:** Mean costs: Physiotherapist vs physician as primary assessor

Cost item	Mean cost (SD)		T-test^**a**^	
	Physiotherapist assessment (€)^**b**^	Physician assessment (€)	Mean difference [95% CI^**c**^]	***p***-value^**d**^
1. Physiotherapist				
*a) Visits*	380 (377)	332 (641)	48 [− 219 to 314]	0.72
*b) Telephone calls*	4.4 (8.2)	0.46 (2.5)	3.9 [0.81 to 7.0]	0.015*
2. Physician				
*a) Visits*	39 (95)	217 (140)	− 178 [− 239 to − 118]	0.000*
*b) Telephone calls*	4.8 (27)	19 (44)	−14 [−33 to 5.5]	0.16
*c) Prescriptions*	5.6 (19)	6.2 (15)	−0.66 [−9.5 to 8.1]	0.88
*d) Letters*	0.79 (4.5)	6.3 (13)	−5.5 [−11 to −0.2]	0.043*
3. Nurse				
*a) Visits*	1.8 (10)	8.0 (34)	−6.2 [−19 to 6.3]	0.32
*b) Telephone calls*	42 (104)	88 (169)	−46 [− 117 to 25]	0.20
4. Radiography	7.9 (25)	32 (42)	−24 [−42 to −6.2]	0.010*
5. Orthopaedic surgeon	22 (85)	33 (100)	−12 [−59 to 36]	0.62
6. Collected prescribed drugs	7.8 (34)	6.6 (16)	1.2 [−13 to 15]	0.87
7. Productivity loss^e^	111 (91)	365 (853)	− 254 [− 728 to 220]	0.27
8. Unpaid work compensation^f^	125 (103)	123 (191)	2.8 [− 113 to 118]	0.96
**Total costs societal perspective**^**g**^	633 (620)	996 (1276)	− 364 [− 891 to 164]	0.17
**Total costs health care perspective**^**h**^	515 (541)	748 (885)	− 233 [− 616 to 150]	0.23

^a^Independent-samples t-test. Dependent variable cost items, independent variable group (physiotherapist or physician assessment)

^b^Euro (€)

^c^Confidence interval

^d^*p*-value, significance level set at *p* < 0.05

^e^Productivity loss for the time the patients were visiting health care or consulting via telephone, including traveling and waiting time. Sick leave days included. Productivity loss was calculated with gross salary including social fees

^f^Unpaid work compensation for the time the patients were visiting health care or consulting via telephone, including traveling and waiting time. Production loss was calculated with net mean salary

^g^Total costs from a societal perspective include all cost items 1–8

^h^Total costs from a health care perspective include cost items 1–6

*Significant, *p* < 0.05

**Table 5:** Results from cost-effectiveness analysis: Physiotherapist vs physician as primary assessor

	Difference in mean costs^**a**^	95% CI^**b**^	Difference in mean QALYs^**c**^	95% CI	ICER^**d**^
**Societal perspective**^**e **^**(*n*** **= 61)**	−364	−870 to 143	−0.015	− 0.093 to 0.063	24,266 €/QALY
**Health care perspective**^**f **^**(*n*** **= 61)**	−233	−605 to 139	−0.015	− 0.093 to 0.063	15,533 €/QALY

^a^Costs are calculated in Euro (€)

^b^Confidence interval

^c^Quality adjusted life years. QALYs were calculated using linear interpolation between each point and using the trapezoidal rule to calculate the “area under the curve”. Presenting β-values from linear regression analysis for group variable adjusted for baseline differences in EQ-5D-3L-index

^d^Incremental cost-effectiveness ratio. Mean difference in costs divided by mean difference in QALYs. Here representing the savings per lost QALY

^e^Societal perspective includes health care visits, prescribed drugs, productivity loss and unpaid work compensation

^f^Health care perspective includes health care visits and prescribed drugs

### Quality adjusted life years

Patients assessed by a physiotherapist or a physician gained 0.75 QALYs respectively 0.74 QALYs 1 year after the initial assessment (MI dataset) and the differences were not significant (*p* = 0.69) (Table 6). When adjusted for baseline differences in EQ-5D-3L index, the mean difference in QALYs were − 0.015 (CI: − 0.093 to 0.063) if the patients were assessed by a physiotherapist first (Table 5).

**Table 6:** Mean QALYs gained after 1 year: Physiotherapist vs physician as primary assessor

Dataset	Mean QALYs (SD)		T-test^**a**^	
	Physiotherapist assessment	Physician assessment	Mean difference [95% CI^**c**^]	***p***-value^**d**^
Complete case	0.65 (0.26)	0.66 (0.23)	−0.009 [− 0.14 to 0.12]	0.88
Last observation carried forward	0.74 (0.17)	0.73 (0.18)	0.009 [−0.074 to 0.093]	0.82
Multiple imputation	0.75*	0.74*	0.015 [−0.059 to 0.089]	0.69

^a^Independent-samples t-test. Dependent variable QALYs, independent variable group (physiotherapist or physician assessment)

^b^Confidence interval

^c^*p*-value, significance level set at *p* < 0.05

*Pooled data from multiple imputations in five different imputed datasets, no standard deviation available for pooled analysis

### Cost-effectiveness

**Fig. 3:**
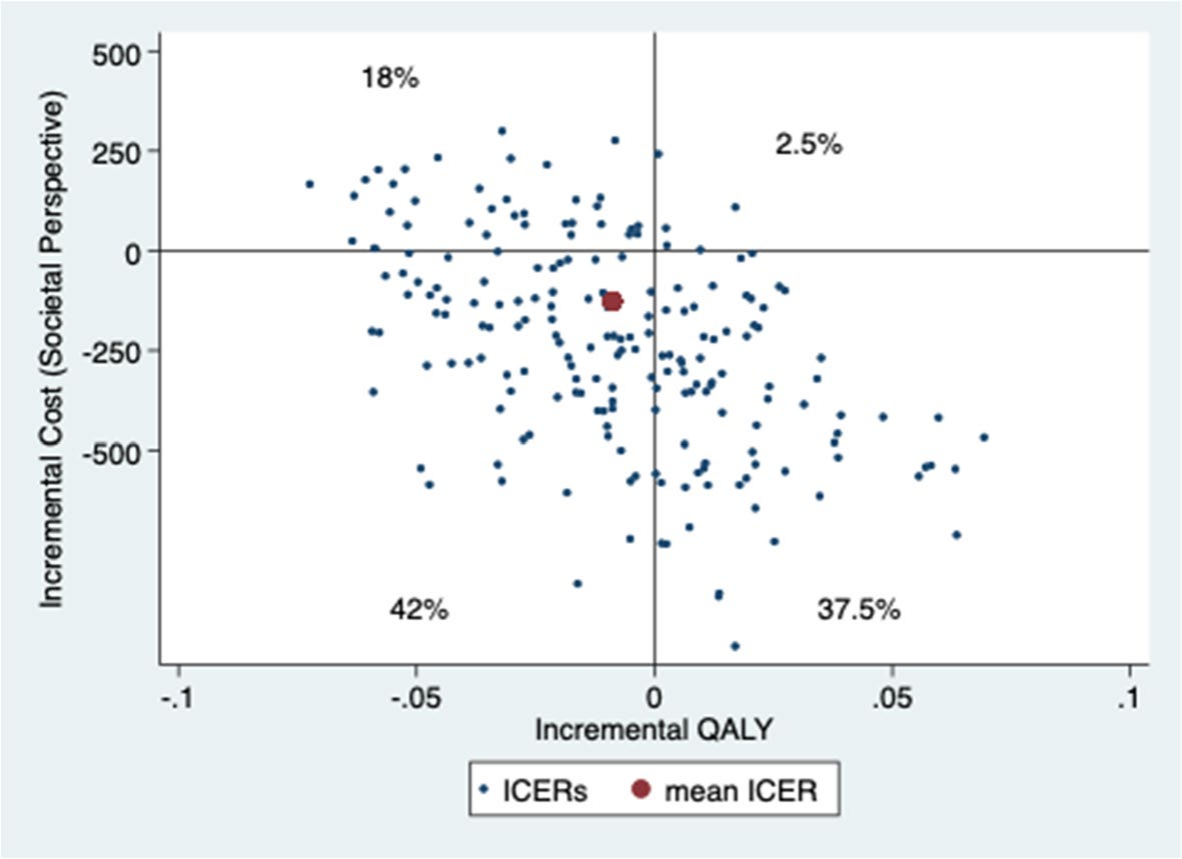
Cost-effectiveness plane societal perspective

**Fig. 4:**
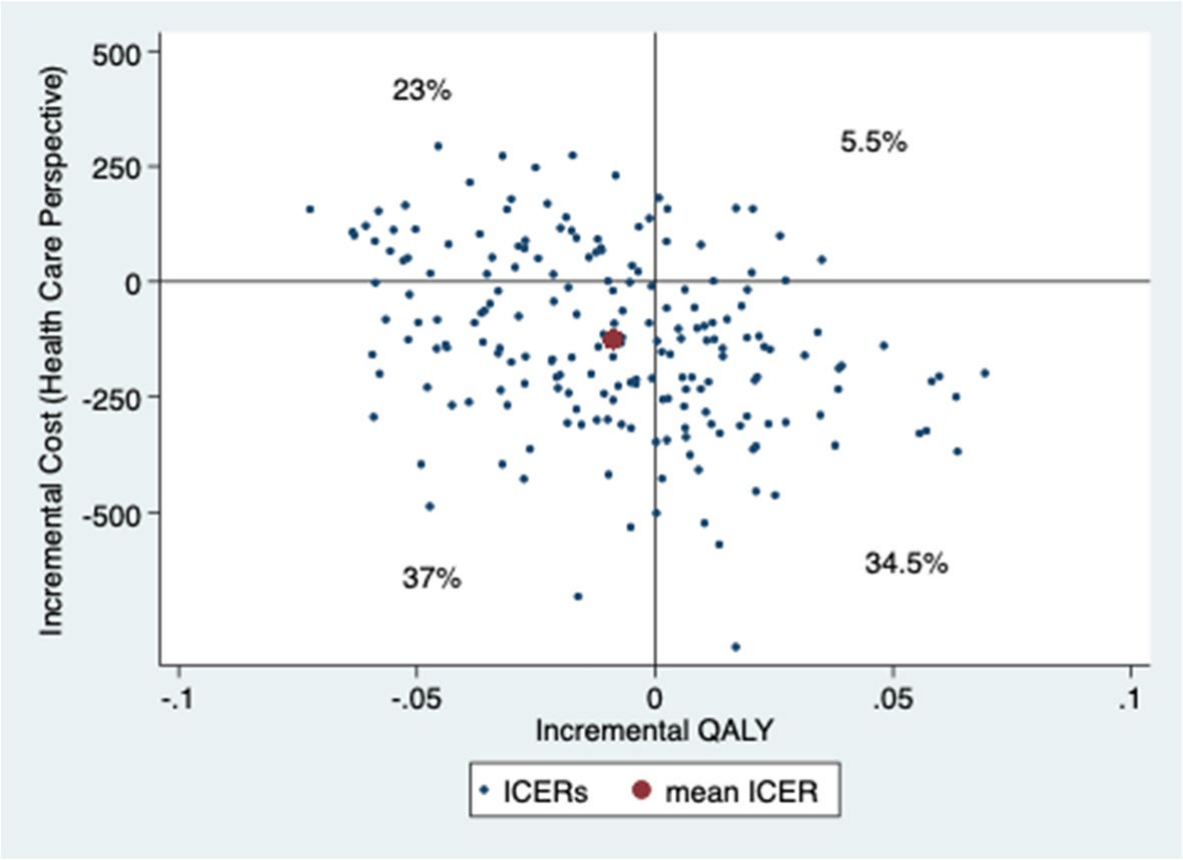
Cost-effectiveness plane health care perspective

**Fig. 5:**
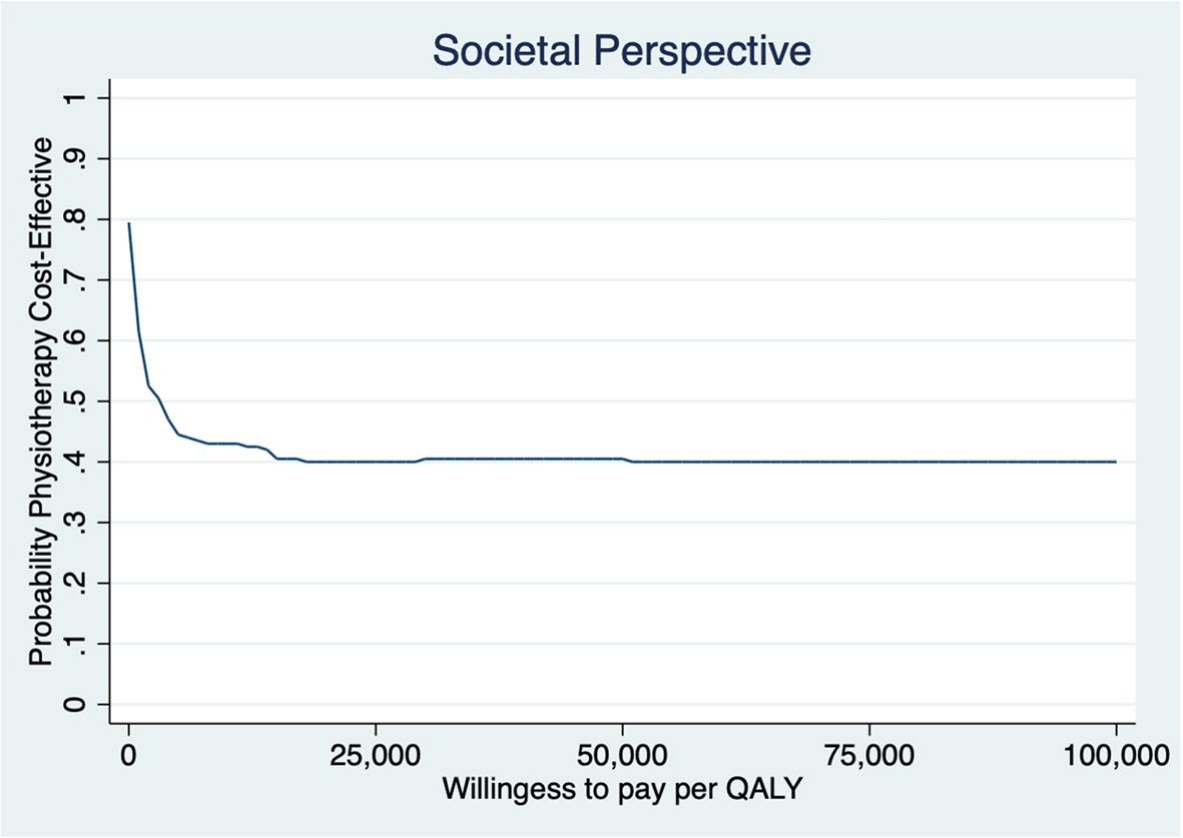
Cost-effectiveness acceptability curve: societal perspective

**Fig. 6:**
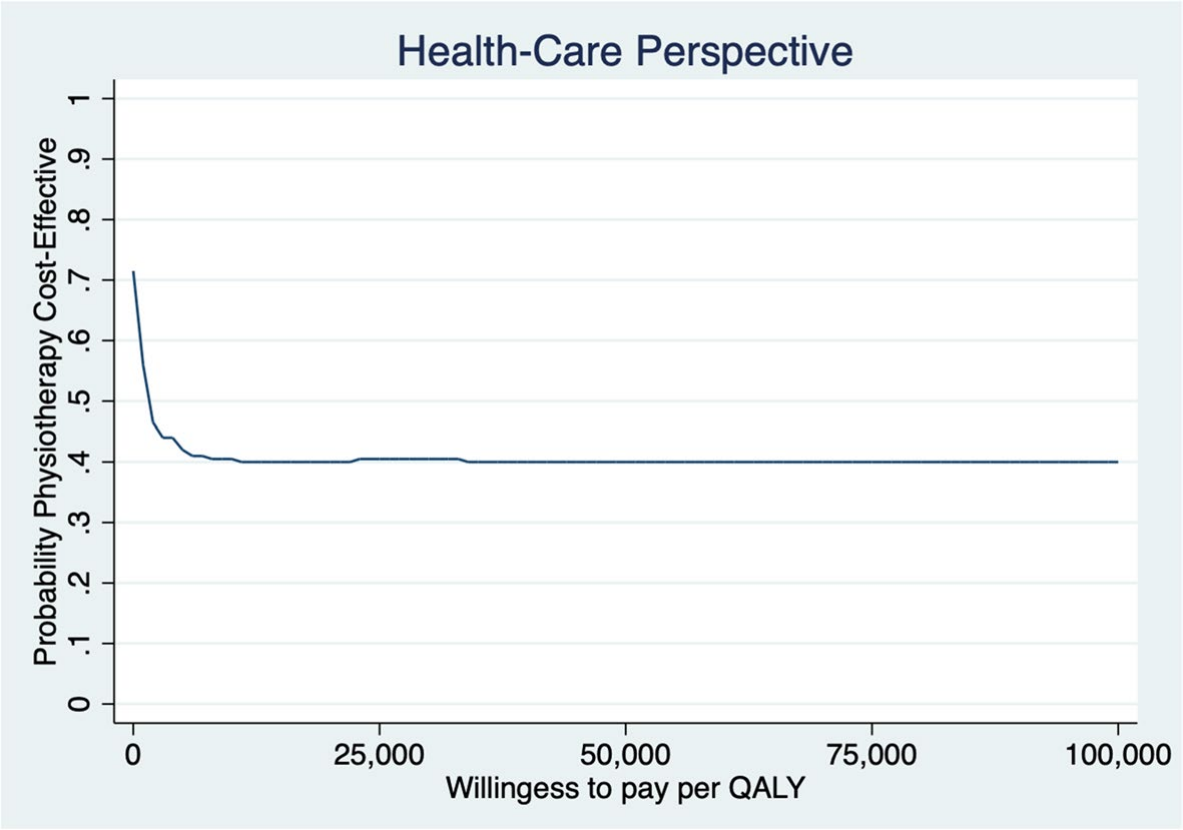
Cost-effectiveness acceptability curve: health care perspective

Patients gained slightly less QALYs but at a lower cost 1 year after the initial contact when assessed by physiotherapist first compared to being assessed by a physician first. Mean ICER of physiotherapist vs physician first from a societal perspective was 24,266 €/lost QALY and from a health care perspective was saving 15,533 €/lost QALY (MI-results) (Table 5) (results of analyses using CC and LOCF are available in Additional file 1). The uncertainty of ICER was analysed with 200 bootstrapped replicates (Figs. 3 and 4). The results from a societal perspective (Fig. 3), where the likelihood that physiotherapist-first leads to lower QALYs at lower costs is 42%, to higher QALYs at lower costs is 38% and to lower QALYs at higher costs is 18%. The point estimate of ICER from a health care perspective and most replicates (37%) are in the lower left quadrant, which represents the likelihood of patients assessed first by a physiotherapist gaining fewer QALYs but at a lower cost than patients first assessed by a physician. However, the likelihood of higher QALYs at lower costs is almost as high (35%), while the likelihood of lower QALYs at higher costs is somewhat lower (23%) (Fig. 4). At the “informal” low threshold value rule from the National Board of Health and Welfare, of ~ 9800 € per QALY (100,000 SEK/QALY) [50], the likelihood that the intervention with physiotherapy as primary assessor is cost-effective is approximately 40% (Figs. 5 and 6).

## Discussion

Our goal was to determine the cost-effectiveness 1 year after assessment for patients with KOA comparing physiotherapists and physicians as primary assessors in primary care. Our findings suggest that physiotherapist-led care model might reduce health care costs and lead to marginally less QALYs, but CIs were wide and overlapped no difference at all.

There were significantly higher costs for physician visits and radiography in the physician group (*p* < 0.001 and *p* = 0.01). According to guidelines, radiography is not required to diagnose patients with typical presentation of OA [24]. However, about one third of the patients assessed by a physician first were referred to radiography. Utilisation of radiography has increased over the past 15 years in Sweden [51] and the costs are 742,000 €/year for patients with OA registered in the national quality registry BOA [52]. Data from the BOA registry show that in 2019, nearly 70% of the patients were referred to radiological examination. Only 5% of all patients in the BOA registry were directly triaged to a physiotherapist [52]. Applying the results from this study to a national setting, like the registry data, would reduce the referral rate to radiography to 9%, if patients were initially assessed by physiotherapists. Consequently, the costs for radiography would decrease by 87%, to 98,200 €/year. Except cost savings, reducing radiography examinations could minimise the ecological footprint with decreased carbon dioxide emissions [53].

It is likely (72–80%) that management of KOA after first assessment by physiotherapist is less costly and differs less than ±0.1 in QALY compared to being assessed by physician first (Figs. 3 and 4), although the CEA-results should be interpreted with caution (Table 5). The results showing improved HrQoL and fewer physician visits at a lower cost compared with traditional physician-led care, are consistent with results from Bornhöft et al. who evaluated the health effects on triaging patients with musculoskeletal disorders to physiotherapists in Swedish primary care [54–56]. However, Bornhöft et al. found that triaging to physiotherapists in primary care has a 85–93% likelihood to be cost-effective at a threshold value of 20,000 € [56] compared with this study that have a likelihood of approximately 40%. The CEAC slopes downwards, which implies that the most likely outcome is that the intervention with physiotherapists as primary assessor results in both lower cost and lower QALYs.

This study has several limitations. The sample size calculation was based on the outcomes in the clinical trial. The sample size of 100 patients was not reached because of low patient flow, which may increase the risk of type II error in this study. This study is based on a pragmatic trial, which may explain the low patient flow. During the study there were organizational changes for both health care centres and rehabilitation centres. Even though pragmatic trials are challenging as they compete with the clinics’ interests, the study design is a strength of this study where this model of care can be easily implemented since the intervention has been tested in a real clinical setting [57]. Pragmatic trials are also thought to provide important information on the optimal delivery of early physiotherapy [58]. Health care visits and costs were not imputed because we cannot be sure of the cause of the missing values, i.e. did the patient consult elsewhere or not at all. The imputed datasets for missing EQ-5D-3L index values resulted in higher total QALYs and the small mean differences shifted from favouring the physician group to the physiotherapist group (Table 6). The small differences remained small, and neither were there any significant differences with the imputed datasets. Despite different MI techniques and using SPSS in the main analyses and STATA in the sensitivity analyses, the MI-results did not differ. A lifetime horizon would be more desirable in economic evaluations of KOA. However, that is a costly and demanding setting, and shorter horizons have been accepted in comparative studies [59]. The current prices for different health care visits are based on regional price lists from the years when the study was conducted, with mean costs, including overhead costs, social fees and patient fees, and time frames based on clinical professionals’ estimations. Mean cost levels used in the CEA were 156 €/physician visit and 63 €/physiotherapist visit, and if we were to use mean salaries and estimated time spent per visit the prices would be 29 € respectively 25 € instead. The mean difference in total costs between the groups would probably be smaller or even be more costly for the physiotherapist group using mean salaries and time, since physiotherapy comprises longer treatment periods with more visits. Another aspect not included in this CEA is the escalating cost for renting physicians and nurses to cover the high demand in Swedish primary care. The cost for renting a physician is estimated to be 2–3 times higher than the cost for own personnel [60, 61]. In Sweden, a medical certificate issued by a physician is required after the first week of work absence. The total productivity loss in this study would be more precise if we also collected data about sick leave periods shorter than 1 week. The small differences in QALY may be a result of the EQ-5D-3L instrument and its risk for ceiling effect and ability to detect change. The EQ-5D with five levels (EQ-5D-5L) reduces the ceiling effect (i.e. reporting no problems in all dimensions) to 30% from 46% with the EQ-5D-3L [62]. The 5 L has better ability to discriminate between milder health problems and identify small changes in health status [63]. Moving from 3 L to 5 L could possibly lower the incremental QALY gain [64–66].

Baseline values of EQ-5D-3L index in the physiotherapist group (0.73) differ from other studies where patients with KOA receiving core treatments had a mean index of 0.60. The 12-month follow-up index value from those studies (mean 0.64) were more similar to the CC values (means 0.65–0.66) than the imputed values (LOCF and MI: 0.73–0.75) [67]. In addition to earlier mentioned factors affecting the EQ-5D-3L index, other aspects may be the inclusion of patients with only mild to moderate symptomatic KOA and the smaller sample size than required in this study.

In line with recommended guidelines, the referral rate was high to physiotherapists in the physician-led pathway, where most patients in the study received recommended treatment of exercise and education. Exercise therapy is an effective treatment to improve health in patients with KOA [68]. Like earlier findings with direct access to a physiotherapist for musculoskeletal disorders, this study resulted in HrQoL improvement and effects comparable to physician-led care [19].

To the best of our knowledge, this study is the first of its kind to evaluate the economic impact of different primary assessors for patients with KOA. This economic evaluation provides support for the increasing use of physiotherapists first for patients with KOA. The knowledge of patients’ perceptions of different primary assessors can be useful when implementing this model of care, and this needs to be explored in future qualitative studies. Physiotherapist as primary assessor could contribute to solving the issue of the estimated increase in KOA physician consultations and costs. In 2019, 44 countries had direct access to physiotherapy [16]. Besides addressing the possibility of direct access to physiotherapy, decision makers must also provide enough physiotherapists in primary care to implement a potential model of care with task-shifting responsibilities.

## Conclusion

These findings suggest that physiotherapist-led care model might reduce health care costs and lead to marginally less QALYs, but CIs were wide and overlapped no difference at all. Health consequences depending on the profession of the first assessor for KOA seem to be comparable for physiotherapists and physicians, which has been shown in the clinical trial which this study is based on [34]. However, larger clinical trials and qualitative studies to evaluate patients’ perception of this model of care are needed.

## Supplementary Information

**Additional file 1.**

## Data Availability

The data sets generated and analysed during the current study are not publicly available due to the General Data Protection Regulation, which means that every participant’s data is confidential, and unauthorized persons have no access to the dataset, but are available from the corresponding author on reasonable request.

## Abbreviations

BOA: Better management of Osteoarthritis
CC: Complete case
CH: Chan-Mei Ho-Henriksson (corresponding author)
CI: Confidence interval
CEA: Cost-effectiveness analysis
CEAC: Cost-effectiveness acceptability curve
CE-plane: Cost-effectiveness plane
CT: Carina A Thorstensson (co-author)
EQ-5D-3L: Euroqol 5 dimension 3 level questionnaire
EQ-5D-5L: Euroqol 5 dimension 5 level questionnaire
€: Euro (currency)
HrQoL: Health-related quality of life
ICER: Incremental cost-effectiveness ratio
KOA: Knee osteoarthritis
LN: Lena Nordeman (co-author)
LOCF: Last observation carried forward
MI: Multiple imputation
MS: Mikael Svensson (co-author)
OA: Osteoarthritis
QALY: Quality adjusted life years
SD: Standard deviation
SPSS: Statistical package for social science
TKA: Total knee arthroplasty
VAS: Visual analogue scale
