# Supplementary material for: Physiotherapist or physician as primary assessor for patients with suspected knee osteoarthritis in primary care – a cost-effectiveness analysis of a pragmatic trial
Source: BMC Musculoskelet Disord. 2022 Mar 17;23:260. doi: 10.1186/s12891-022-05201-3 (PMC8932301; doi:10.1186/s12891-022-05201-3)
Supplement: Supplementary file 1 — Additional file 1. [file 12891_2022_5201_MOESM1_ESM.docx]

| **Additional file 1.** Results from cost-effectiveness analysis of different datasets: Physiotherapist vs physician as primary assessor | | | | | | |
| --- | --- | --- | --- | --- | --- | --- |
|  | **Difference in mean cost^a^** | **95% CI^b^** | **Dataset for QALY^c^ analysis** | **Difference in mean QALYs^d^** | **95% CI** | **ICER^e^** |
| **Societal perspective^f^**  **(n=61)** | -364 | -870 to 143 | Complete case (n=59) | -0.014 | -0.15 to 0.12 | 26 000 €/QALY |
|  |  |  | LOCF (n=69) | -0.030 | -0.11 to 0.052 | 12 133 €/QALY |
|  |  |  | MI (n=69)*5 | -0.015 | -0.093 to 0.063 | 24 266 €/QALY |
| **Health care perspective^g^ (n=61)** | -233 | -605 to 139 | Complete case^h^ (n= 59) | -0.014 | -0.15 to 0.12 | 16 643 €/QALY |
|  |  |  | LOCF^i^ (n=69) | -0.030 | -0.11 to 0.052 | 7 766 €/QALY |
|  |  |  | MI^j^ (n=69)*5 | -0.015 | -0.093 to 0.063 | 15 533 €/QALY |
| ^a^ Costs are calculated in Euro (€).  ^b^ Confidence interval.  ^c^ Quality adjusted life years. QALYs were calculated using linear interpolation between each measurement point and using the trapezoidal rule to calculate the “area under the curve”.  ^d^ Presenting β-values from linear regression analysis for group variable adjusted for baseline differences in EQ-5D-3L-index.  ^e^ Incremental cost-effectiveness ratio. Mean difference in cost divided by mean difference in QALYs.  ^f^ Societal perspective: include health care visits, prescribed drugs, productivity loss and unpaid work compensation.  ^g^ Health care perspective: include health care visits and prescribed drugs.  ^h^ Complete case analysis, using original dataset without imputed data for EQ-5D-3L index when calculating total QALYs gained.  ^i^ Imputed dataset using last observation carried forward for the EQ-5D-3L index when calculating total QALYs gained.  ^j^ Imputed dataset using multiple imputation in SPSS with regression analysis to predict imputed values for EQ-5D-3L index when calculating total QALYs gained. Presented with pooled data for five different imputed datasets or the range of p-values. | | | | | | |
